# Formulation, evaluation and bioactive potential of *Xylaria primorskensis* terpenoid nanoparticles from its major compound xylaranic acid

**DOI:** 10.1038/s41598-018-20237-z

**Published:** 2018-01-29

**Authors:** Mohd Adnan, Mitesh Patel, Mandadi Narsimha Reddy, Eyad Alshammari

**Affiliations:** 1 Department of Clinical Laboratory Sciences, College of Applied Medical Sciences, University of Ha’il, Ha’il, P.O. Box 2440, Saudi Arabia; 2 Department of Biosciences, Bapalal Vaidhya Botanical Research Centre, Veer Narmad South Gujarat University, Surat, Gujarat India; 3 Department of Clinical Nutrition, College of Applied Medical Sciences, University of Ha’il, Ha’il, P.O. Box 2440, Saudi Arabia

## Abstract

In recent years, fungi have been shown to produce a plethora of new bioactive secondary metabolites of interest, as new lead structures for medicinal and other pharmacological applications. The present investigation was carried out to study the pharmacological properties of a potent and major bioactive compound: xylaranic acid, which was obtained from *Xylaria primorskensis* (*X*. *primorskensis*) terpenoids in terms of antibacterial activity, antioxidant potential against DPPH & H_2_O_2_ radicals and anticancer activity against human lung cancer cells. Due to terpenoid nature, low water solubility and wretched bioavailability, its pharmacological use is limited. To overcome these drawbacks, a novel xylaranic acid silver nanoparticle system (AgNPs) is developed. In addition to improving its solubility and bioavailability, other advantageous pharmacological properties has been evaluated. Furthermore, enhanced anticancer activity of xylaranic acid and its AgNPs due to induced apoptosis were also confirmed by determining the expression levels of apoptosis regulatory genes p53, bcl-2 and caspase-3 via qRT PCR method. This is the first study developing the novel xylaranic acid silver nanoparticle system and enlightening its therapeutic significance with its improved physico-chemical properties and augmented bioactive potential.

## Introduction

Need for novel and beneficial natural products to provide aid and relief in all aspects of human disease conditions is ever growing and even challenging. Throughout the history of drug development, nature has proven and continues to be a promising source for the discovery of bioactive compounds, important for the development of new pharmaceuticals for fighting against infection, inflammation, cancer and various other diseases^1–3^. Fungi have been recognised as a large unexploited source of potentially powerful new pharmaceutical products for the development of medicines and nutraceuticals since ages^4–6^. Though, fungi is an immense source of biological active components, yet, less than ten percent of all species have been described and even less have been tested for therapeutic significance^7,8^.

*X*. *primorskensis* is a fungus of genus *Xylaria*, belongs to the family Xylariaceae. *Xylaria* species are widespread from temperate to the tropical zones of the earth^9^. Phytochemically, fungi of the genus *Xylaria* are quite diversified with respect to their chemical constituents and known to produce diverse classes of bioactive compounds including terpenoids^10–14^, xanthones^15,16^, cytochalasins^17^, cyclopeptides^18,19^, polyketides^20,21^, xyloketals^18^, antifungal metabolites multiplolides A and B^20^, NPY Y5 receptor antagonists xyarenals A and B^10^, polypropionates like xylarinic acids A and B^22^. However, there are no reports on the isolation and characterization of chemical compounds from the *X*. *primorskensis* and its bioactive potential.

In the present study, xylaranic acid, a major terpenoid bioactive compound was obtained from *X*. *primorskensis* and studied for its bioactive potential. This study proves xylaranic acid as a potent pharmacological agent, but its terpenoid nature limited its pharmacological use due to its lower water solubility and pitiable bioavailability. Therefore, a novel AgNPs of xylaranic acid was developed to improve its solubility and bioavailability. Prepared xylaranic acid AgNPs were characterized in order to confirm the synthesis and morphology. Both xylaranic acid and its AgNPs were compared for their antibacterial activity against *Staphylococcus aureus*, *Salmonella typhi* & *Shigella flexneri*, antioxidant potential against DPPH & H_2_O_2_ radicals and anticancer activity against human lung cancer cells.

## Results

### **Identification of*X***. ***primorskensis*Y**. **M**. **Ju**, **H**. **M**. **Hsieh**, **Lar**. **N**. **Vassiljeva & Akulov**

**Figure 1:**
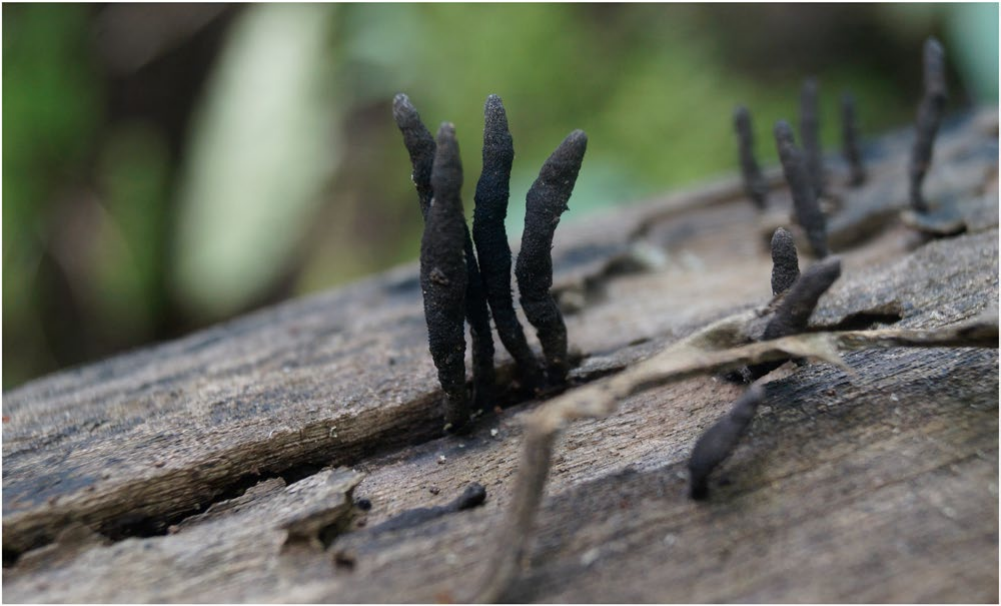
Fruiting body of Xylaria primorskensis in a natural habitat.

On the basis of morphological as well as internal transcribed spacer (ITS) gene sequence analysis, the fungal strain was identified as *X*. *primorskensis* Y. M. Ju, H. M. Hsieh, Lar. N. Vassiljeva & Akulov (Fig. 1). The ITS sequence examination uncovered that the fungal strain had more than 95% grouping closeness with those strains acquired from GenBank. After the successful identification, nucleotide sequences were deposited to NCBI with accession numbers MG012860.

### Phylogenetic analysis

**Figure 2:**
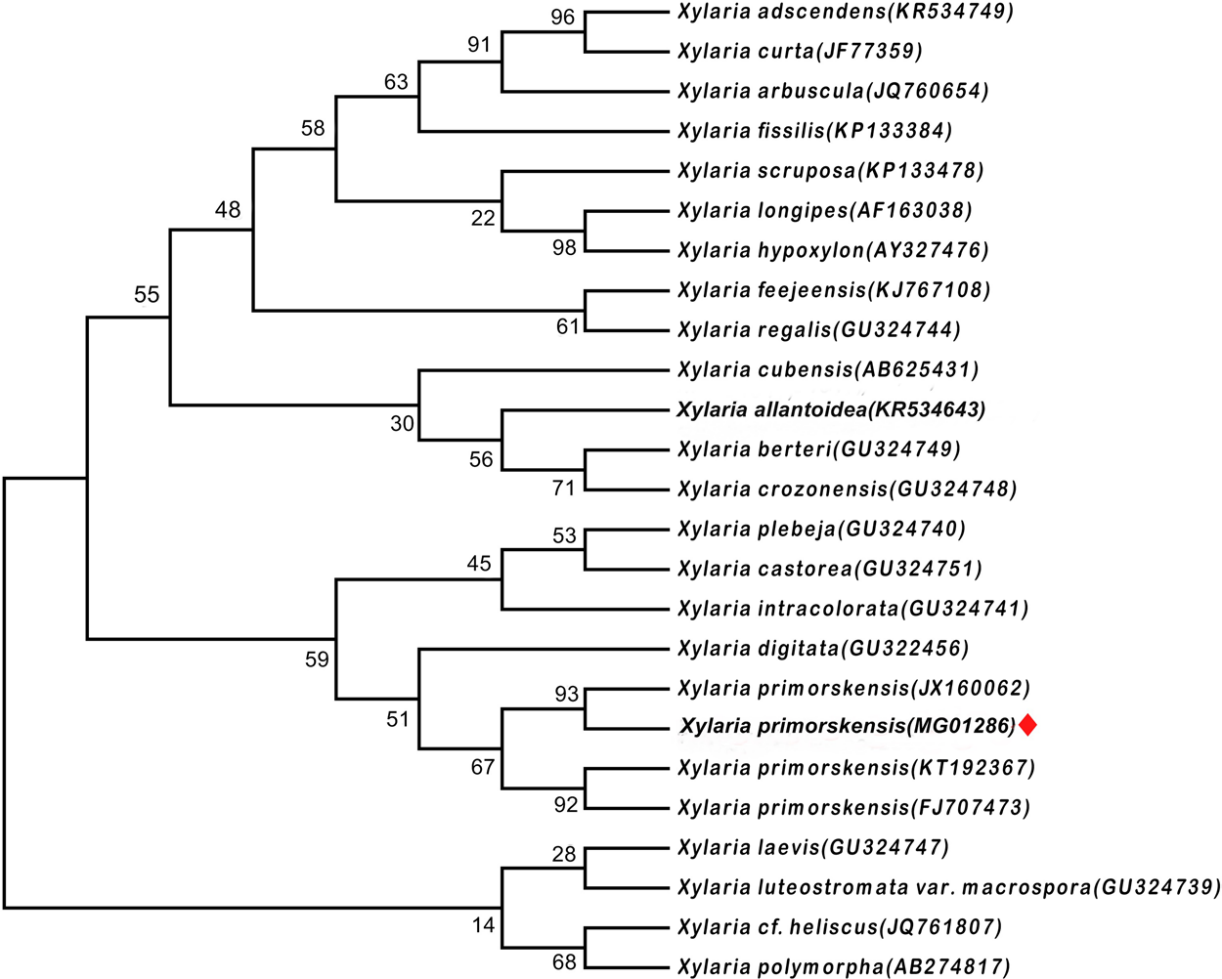
One of the most maximum likelihood tree found by the analysis of ITS1/ITS2 gene sequences of *X*. *primorskensis* and other related species. Bootstrap values are presented above the branches.

The MP tree was obtained using the Subtree-Pruning-Regrafting (SPR) in which the initial trees were obtained with heuristic searches of 1,000 replicates of random stepwise sequence addition. Phylogenetic analysis consisted of bootstrap resampling method of Felsenstein. The bootstrap consensus tree inferred from 1000 replicates, taken to represent the evolutionary history of the taxa analyzed. Maximum likelihood approach was based on the Tamura-Nei model in Molecular Evolutionary Genetics Analysis (MEGA 7.0), followed by 1000 replicates. Our sequence of *X*. *primorskensis* was found to placed in the clade of *X*. *primorskensis* (Fig. 2).

### Terpenoids extraction and identification

**Figure 3:**
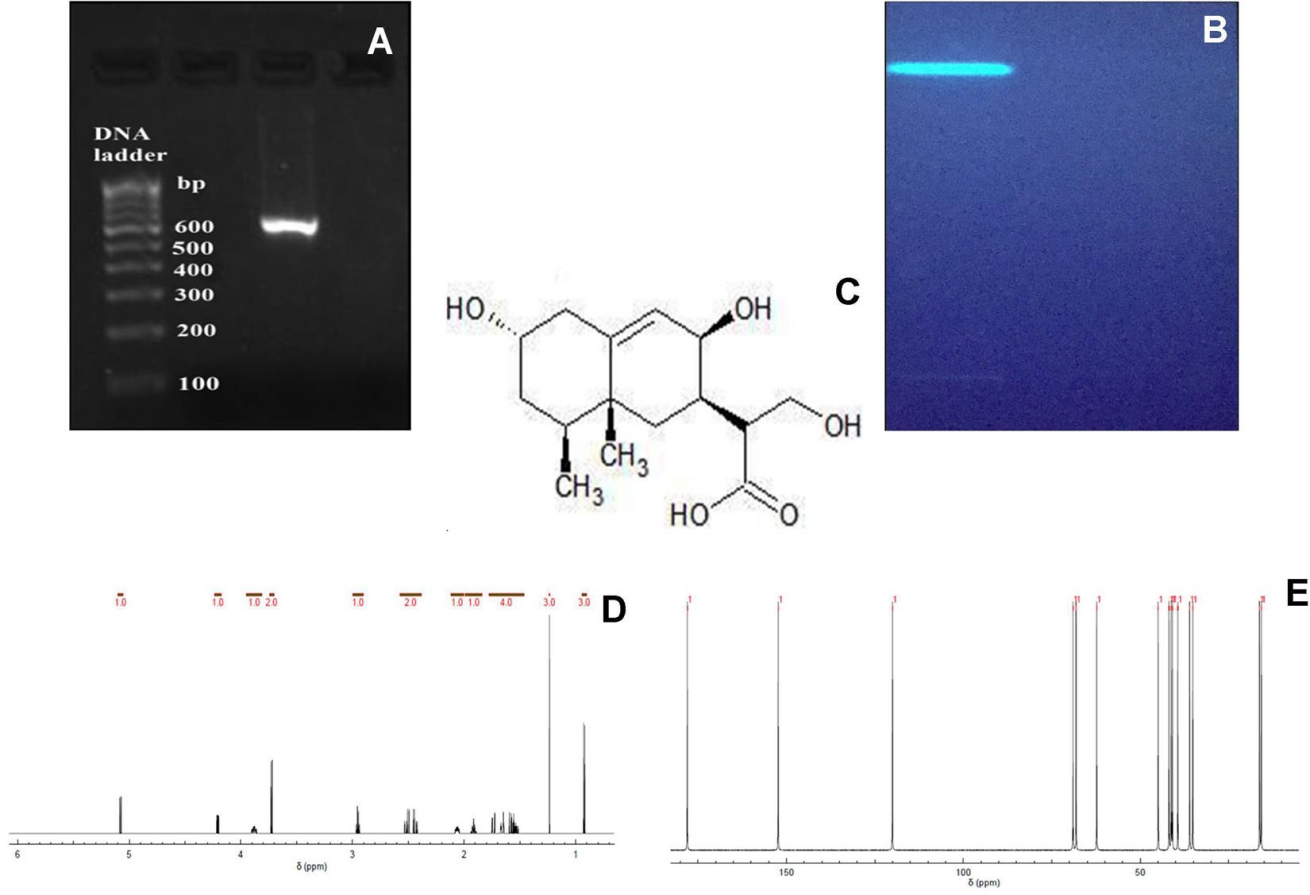
(**A**) Amplification of *X*. *primorskensis* ITS region (BAB 5000-Sample ID). (**B**) HPTLC analysis of purified terpenoids fraction extracted from the *X*. *primorskensis* observed under UV light at 365 nm (bright) range. (**C**) Structure of Xylaranic acid. (**D**) ^1^H-NMR spectra^*^. (**E**) ^13^C-NMR spectra^*^. *Chemical shift values can be seen in detail as supplementary data S1.

Crude terpenoids were extracted from *X*. *primorskensis* powder by solvent extraction method. Extracted crude terpenoids were qualitatively confirmed by Salkowski’s test and High Performance Thin Layer Chromatography (HPTLC) method. In Salkowski’s test, terpenoids appeared as reddish brown colour at the inner face. Whereas, HPTLC profiling revealed the presence of 3 compounds having different Rf values in Benzene: Ethyl acetate (1:1) solvent system. Extracted crude terpenoids were further subjected to a column of silica gel and eluted with gradually increasing polarity of hexane/ethyl acetate (0 to 100%) followed by 100% methanol. Total 7 fractions were collected in which similar fractions were pooled in each other. Finally, 3 terpenoid fractions were collected which screened for their antibacterial potential against *Staphylococcus aureus* (MTCC 3160). Out of them, most potent antibacterial fraction number 3 was identified as xylaranic acid elucidated by spectroscopic methods and the ^1^H and ^13^C NMR spectral data (Supplementary Data S1) were compared with earlier reports^23^ (Fig. 3C–E).

### Depiction of xylaranic acid AgNPs

**Figure 4:**
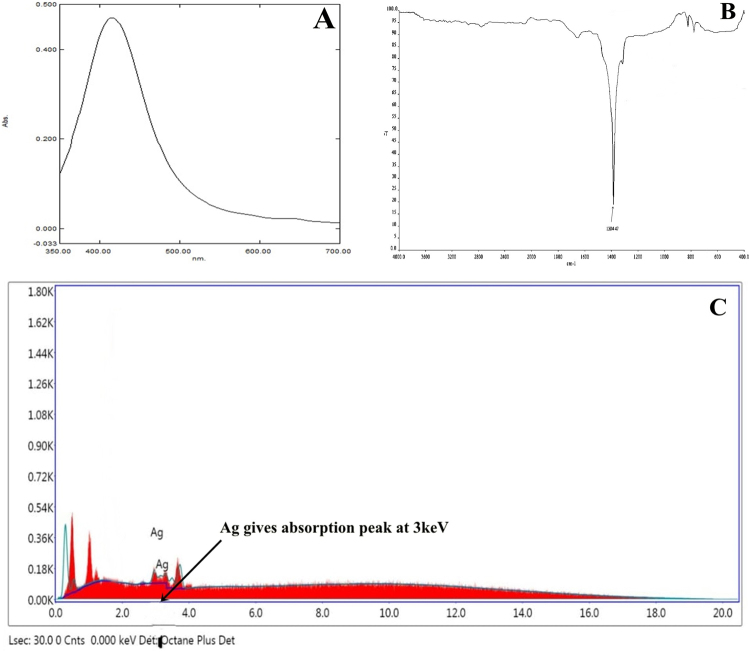
(**A**) UV-Visible absorption spectra of xylaranic acid AgNPs. (**B**) FTIR analysis of xylaranic acid AgNPs. FTIR peak at 1384.87 cm^−1^ indicates the –OH group stretching indicating the involvement in reduction of silver ions. (**C**) Energy dispersive spectra of the xylaranic acid AgNPs.

For the determination of formation of silver nanoparticles into the aqueous solutions, UV-visible spectroscopy is an important and most widely used technique. The formation and constancy of synthesized xylaranic acid AgNPs were primarily examined by UV–Vis analysis. Spectroscopy measurements performed after 24 hours in which absorption spectra of synthesized xylaranic acid AgNPs showed decidedly symmetric single-band absorption with peak maximum at 412 nm (Fig. 4A). This point out the presence of xylaranic acid AgNPs which is due to the excitation of surface plasmons. However, spectroscopy measurements were also performed after two weeks of synthesis and there was no discernible variation in the spectroscopic results of the synthesized nanoparticles, which indicates their stability.

Furthermore, Fourier Transform Infrared Spectroscopy (FTIR) analysis was also carried out to assess the possible molecular interactions between xylaranic acid and AgNO_3._ As shown in figure, absorption band at 1384.87 cm^−1^ shows the OH deformations in the xylaranic acid due to the formation of nanoAg (Fig. 4B). This indicates that AgNO_3_ formed molecular bonds with the OH functional group on xylaranic acid with intermolecular hydrogen bonds.

**Figure 5:**
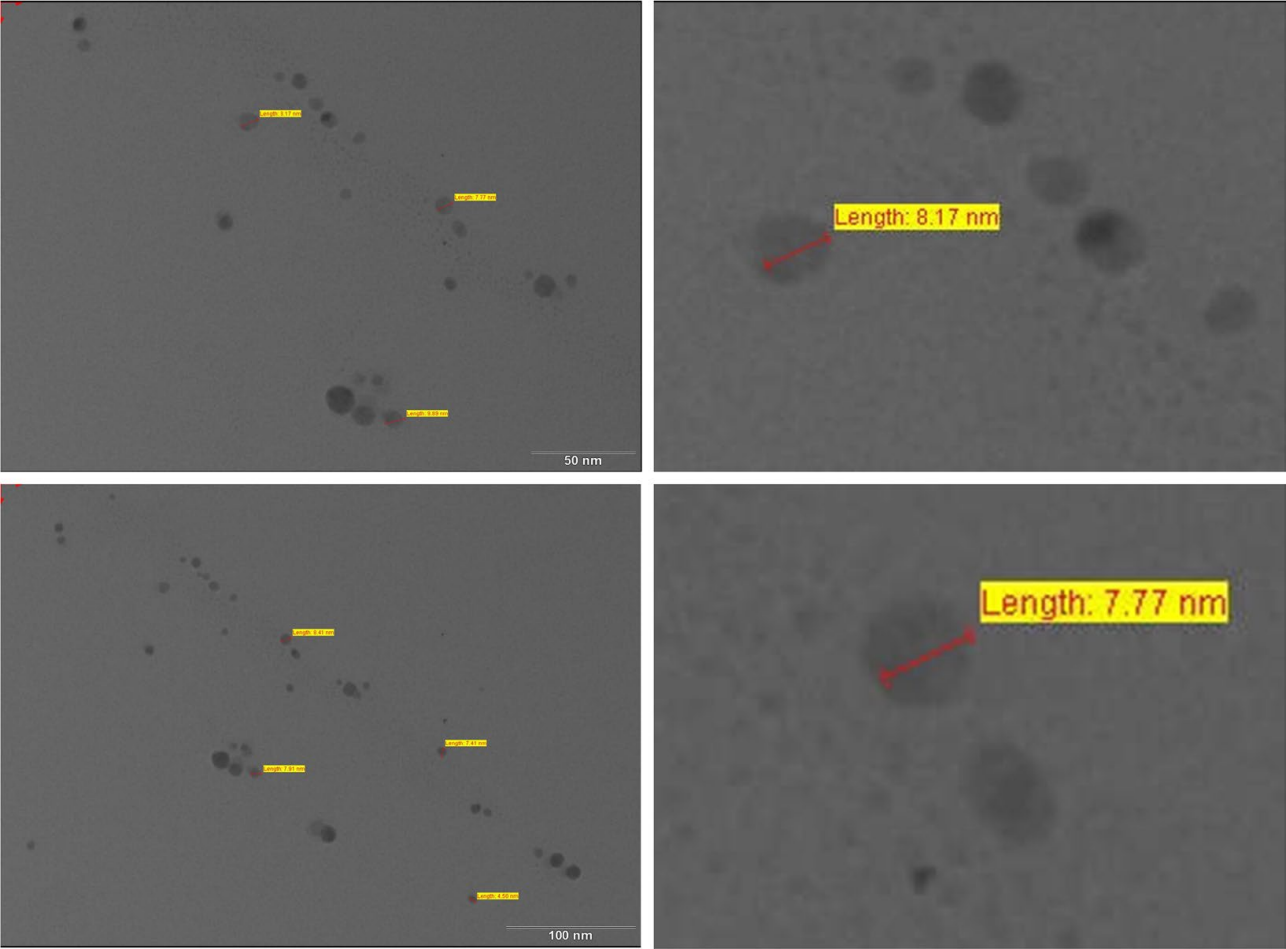
TEM micrograph showing morphological analysis of xylaranic acid AgNPs with variable diameter. Particle size is found to be in the range of 4.50–17.75 nm.

Energy Dispersive Analysis X-Ray spectroscopy (EDS) spectra was also recorded from the synthesized xylaranic acid AgNPs and is shown in Fig. 4C, which confirmed the reduction of silver ions into elemental silver. EDS profile shows the optical absorption peak of elemental silver nanocrystals approx at 3 KeV along with weak copper and carbon peaks from the grid used. Morphological analysis of synthesized xylaranic acid AgNPs was conceded out using Transmission Electron Microscopy (TEM) (Fig. 5). TEM micrograph revealed the size and shape of the synthesized xylaranic acid AgNPs. Xylaranic acid AgNPs were small enough to be electron transparent and looks like spherical shape with variable diameter. Figure 5 shows the, particle size is in the range of 4.50–17.75 nm.

### Antibacterial potential and Antioxidant property of Xylaranic Acid and its synthesized AgNPs

**Figure 6:**
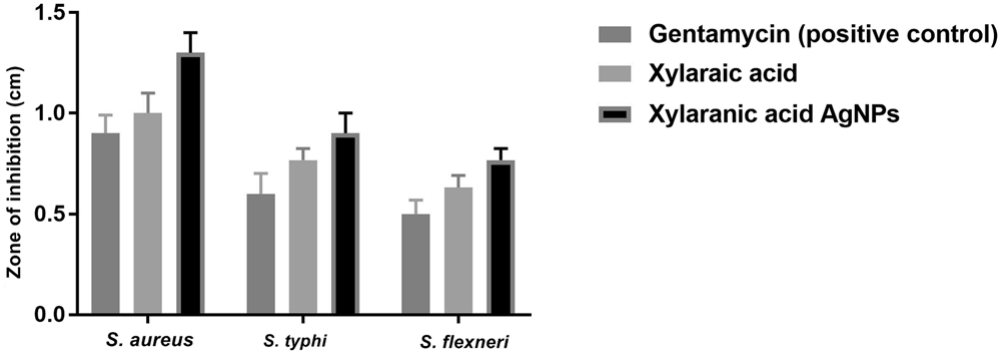
Antibacterial assay of xylaranic acid and xylaranic acid AgNPs against *S*. *aureus*, *S*. *typhi* and *S*. *flexneri*. Error bars represent SD of the mean values of results from three replicate experiments.

Xylaranic acid & synthesized xylaranic acid AgNPs were studied for their antagonistic potential against pathogenic bacteria like *S*. *aureus*, *S*. *typhi* and *S*. *flexneri*. Results of antibacterial activity are represented in the form of zone of inhibition (Fig. 6 and Supplementary Figure S2). Synthesized xylaranic acid AgNPs has higher antagonistic activity compare to xylaranic acid. Both xylaranic acid and xylaranic acid AgNPs showed higher activity against *S*. *aureus* when compared to *S*. *typhi* and *S*. *flexneri*.

**Figure 7:**
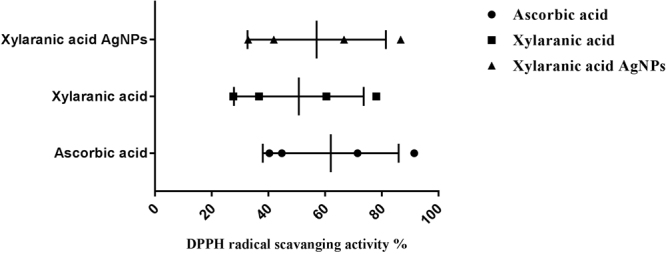
Antioxidant potential of xylaranic acid and xylaranic acid AgNPs against DPPH free radical. Error bars represent SD of the mean values of results from three replicate experiments.

**Figure 8:**
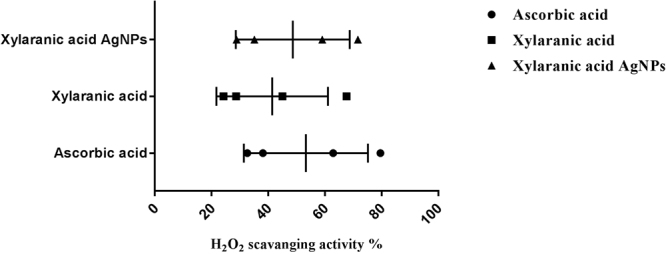
Antioxidant potential of xylaranic acid and xylaranic acid AgNPs against H_2_O_2_ molecule. Error bars represent SD of the mean values of results from three replicate experiments.

Antioxidant potential was studied against DPPH and H_2_O_2_ molecules in comparisons to ascorbic acid. Both xylaranic acid and xylaranic acid AgNPs exhibited good radical scavenging capacity against both DPPH and H_2_O_2_ molecules. Xylaranic acid AgNPs were better DPPH and H_2_O_2_ scavengers compared to xylaranic acid. Both xylaranic acid and xylaranic acid AgNPs reflected dose dependence of the antioxidant potentials as there was increase in their concentration (25 µg/ml, 50 µg/ml, 75 µg/ml and 100 µg/ml), antioxidant potential was also increased (Figs 7 and 8).

### Cytotoxic activity with expression level of apoptosis related genes

**Figure 9:**
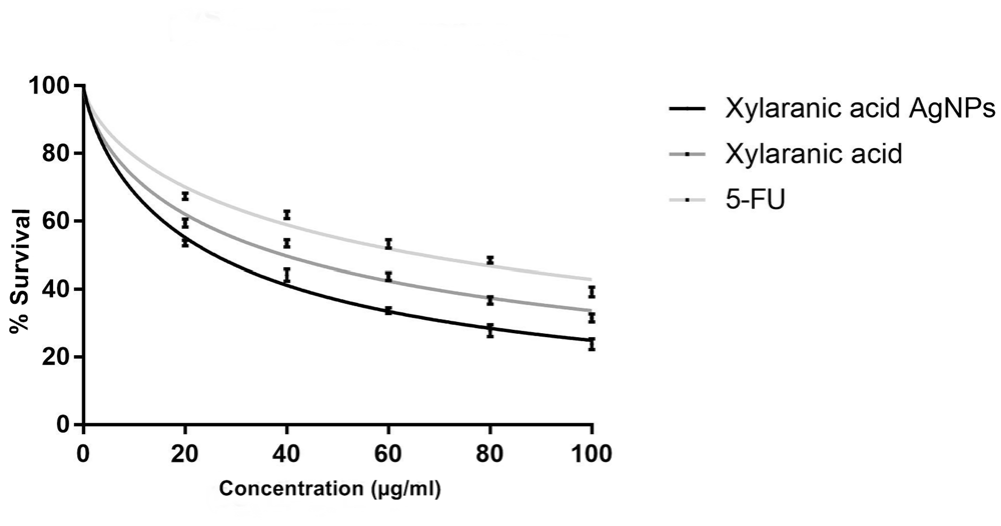
Cytotoxic activity of xylaranic acid and xylaranic acid AgNPs against A549 cell line. Activity of xylaranic acid AgNPs was found to be higher than the xylaranic acid. The effects of xylaranic acid and its AgNPs on lung cancer cells can also be seen (Supplementary Figure S3).

Different concentrations of xylaranic acid and xylaranic acid AgNPs were tested for their cytotoxic effect against A549 cells. Both compounds were tested at different concentrations (20, 40, 60, 80, and 100 µg/ml) under comparable conditions. Both xylaranic acid and xylaranic acid AgNPs showed dose-dependent cytotoxic activity on A549 cell lines and IC 50 values of xylaranic acid and synthesized xylaranic acid AgNPs were, respectively, 39.39 and 25.84 µg/ml for A549 cancer cell line. Cytotoxic activity of xylaranic acid AgNPs was higher than the xylaranic acid (Fig. 9). The effects of xylaranic acid and its AgNPs on lung cancer cells can also be seen (Supplementary Figure S3).

**Figure 10:**
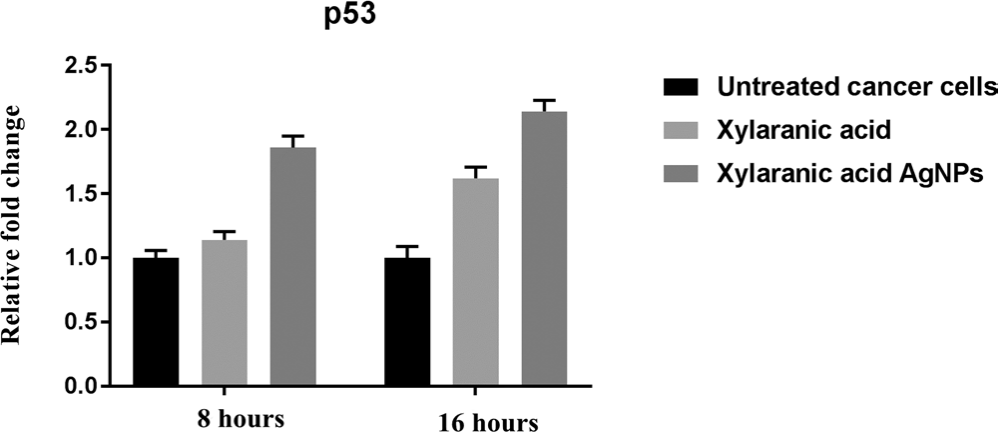
Effect of xylaranic acid and xylaranic acid AgNPs at IC 50 values for 8 and 16 hours incubation on expression level of p53. Statistical analysis by two-stage linear step-up procedure of Benjamini, Krieger and Yekutieli, with Q = 1%.

**Figure 11:**
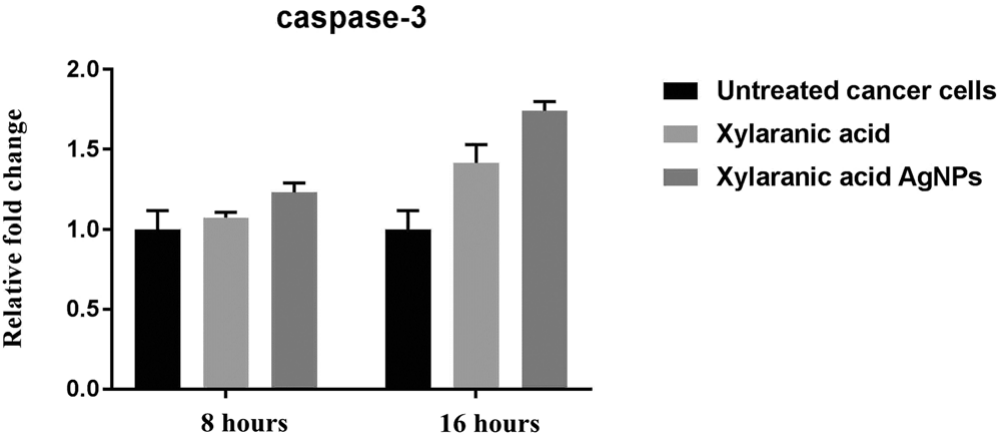
Effect of xylaranic acid and xylaranic acid AgNPs at IC 50 values for 8 and 16 hours incubation on expression level of caspase-3. Statistical analysis by Two-stage linear step-up procedure of Benjamini, Krieger and Yekutieli, with Q = 1%.

**Figure 12:**
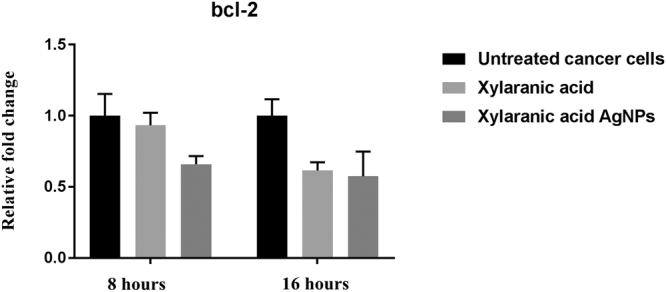
Effect of xylaranic acid and xylaranic acid AgNPs at IC 50 values for 8 and 16 hours incubation on expression level of bcl-2. Statistical analysis by Two-stage linear step-up procedure of Benjamini, Krieger and Yekutieli, with Q = 1%.

Expression levels of apoptosis-related genes p53, bcl2 and caspase-3 in A549 lung cancer cells which were induced by xylaranic acid and xylaranic acid AgNPs were determined by real time PCR. Expression level of p53 and caspase-3 genes were increased in both cells treated with xylaranic acid and xylaranic acid AgNPs after 8 and 16 hours incubation compared to untreated cells. The expression level of both apoptosis-related genes were time dependent (Figs 10 and 11). Whereas, expression level of bcl2 gene was decreased in both cells treated with xylaranic acid and xylaranic acid AgNPs after 8 and 16 hours incubation compared to untreated cells (Fig. 12).

## Discussion

Terpenoids are the largest group of natural compounds, with more than 25,000 individual compounds identified to date which have different kinds of biological activities and used for the treatment of human diseases. Terprnoids display a wide range of biological activities like anti-cancer activity, anti-malarial activity, anti-inflammatory activity, anti-bacterial activity, anti-viral activity etc. Terpenoids based pharmaceutical products comprises a multi-million-dollar market worldwide^24^. Among these, Artemisinin and Taxol are the most renowed terpene based drugs. Artemisinin, is a phytoconstituent isolated from *Artemisia annua* L. which possesses antimalarial activity^25^.

Tanshinones is a diterpene which was extracted from the *Salvia miltiorrhiza* which demonstrate a potent anti-cancer activity against various cancer cells like colon, stomach, prostate, breast, liver, lung and leukemia^26^. Another diterpene isolated from *Euphorbia peplus* L. is Ingenol 3-angelate which also exhibited anti-cancer activity against various cancer cell lines, including malignant melanoma that are resistant to conventional therapeutic agents^27^. Ginkgolides (diterpenes) and bilobalide (sesquiterpenes) extracted from *Ginkgo biloba* has been used for a long time for improvement of blood circulation and strengthening of the vessel system^28^. Terpinen-4-ol- a major compound of tea tree oil has been used as a traditional medicine as a strong antimicrobial agent against various different pathogenic bacteria^28^. Betulinic acid (BA) is a pentacyclic triterpene, which recognize as an effective against HIV through the inhibition of replication^29^. Ursolic acid (UA) is a pentacyclic carboxylic acid present in medicinal herbs which demonstrates bioactivities ranging from anti-inflammatory, antiproliferative, proapoptotic, antimetastatic to antiangiogenic, reported in both *in vitro* and *in vivo*^30^.

To date, many structurally-novel terpenoid compounds have been isolated from different living organisms like plants, fungi, marine bacteria, sponges, molluscans etc^30–32^ and no doubt that more terpenoid compounds will become available in the near future as a potent bioactive agent in the treatment of different human diseases. Xylaranic acid is a fungal terpenoid which is normally present in the fungi of *Xylaria* genus. This study is the first one which revealed the wide spectrum medicinal properties of xylaranic acid such as antibacterial, antioxidant and anticancer which have been not known previously. As xylaranic acid is the medicinally important compound in terms of antibacterial, antioxidant and anticancer activity, we performed further investigations with this compound. Xylaranic acid belongs to a class of terpenoids, which has poor solubility in water and dissolution properties which may be lead to poor oral bioavailability and thereby bound its future developments for medicinal applications. In recent times, nanotechnology emerged as a highly promising technology to get better drug’s water solubility and enhanced delivery^34–37^.

Nanoparticles are stable colloidal particles of a size ≤100 nm and their use in medicine and more specifically drug delivery is set to spread rapidly, specifically for cancer therapy^38^. Due to difficultly in silver nanoparticle synthesis, compact stability and former concern about silver toxicity, traditionally it has been not applied in drug delivery applications. However, recent clinical use of silver nanoparticles in wound care as an effective antimicrobial solution as well as recent *in vivo* studies providing positive safety assessments for systemic exposures have encouraged biomedical research with silver nanoparticles. Furthermore, recent improvements in silver nanoparticles biocompatibility via surface modification, as well as exceptional optical properties have also improved suitability of silver nanoparticles for drug delivery^39,40^.

Synthesis of xylaranic acid AgNPs was observed when the isolated xylaranic acid was incubated with the AgNO_3_ solution. Synthesized xylaranic acid AgNPs exhibit yellowish brown colour due to excitation of surface plasmon vibrations in silver nanoparticles. This change in colour indicated that there is a direct correlation between the colour and concentration of the reducing agents such as terpenoids^41^. Formation of xylaranic acid AgNPs is assumed to be in accordance with ‘LaMer Model’^42^. According to that model, reduction of Ag^+^ ions is followed by condensation and surface reduction to AgNPs. Similarly, like previous studies on silver nanoparticles formation from plant terpenoids, AgNPs formation from fungal terpenoids also contribute same absorption bands at around 400–420 nm in the UV-vis spectra. UV-vis absorption spectra of xylaranic acid AgNPs showed that the broad surface plasmon resonance at 412 nm (Fig. 3A). TEM analysis revealed the surface morphology and size of xylaranic acid AgNPs. Xylaranic acid AgNPs predominates with spherical in shape with size ranging from 4.50–17.75 nm (Fig. 4). FTIR peak at 1384.87 cm^−1^ indicates the –OH group stretching indicating the involvement in reduction of silver ions.

The antibacterial activity of xylaranic acid and xylaranic acid AgNPs has also been investigated in this study. In comparisons to xylaranic acid, xylaranic acid AgNPs clearly depicts the enhanced antagonistic activity. Higher antagonistic activity of xylaranic acid AgNPs might be considered due to the sturdy association of AgNPs with the cell wall of the pathogenic bacteria which may effect in the formation of pits, affecting the permeability and finally cells undergoes in death^43^. According to Hard soft acids bases (HSAB) theory, interaction of Ag^+^ ions with phosphorus of phosphate molecule of DNA and similarly interaction of Ag^+^ ions with sulphur of cysteine residues of proteins results into the breakdown of mitochondrial function, denaturation of protein, damaging of DNA and ultimately leads to the bacterial cell death^44–47^. However, ESR studies also revealed the antagonistic activity of AgNPs due to the generation of free radicals from AgNPs^48^.

Antioxidant potential study revealed xylaranic acid AgNPs were better DPPH and H_2_O_2_ radical scavengers than the xylaranic acid. Higher antioxidant potential of xylaranic acid AgNPs due to the presence of xylaranic acid and silver ions could result to antioxidant activities proceeding through hydrogen atom transfer (HAT) and single electron transfer (SET) mechanisms simultaneously^49^. Antioxidant potential of xylaranic acid AgNPs recommend their use in the prevention of different oxidative stress associated with degenerative diseases as natural antioxidant.

In order to determine whether the xylaranic acid and synthesized xylaranic acid AgNPs possesses anticancer activity, we also performed cell viability studies in human lung cancer cells. In particular, xylaranic acid AgNPs showed more potent anticancer effect on lung cancer cells compared to xylaranic acid. To elucidate the molecular mechanisms for the antiproliferative effect of synthesized xylaranic acid AgNPs on human lung cancer cells, we investigated the mRNA expression level of genes (p53, bcl-2 and caspase-3) involved into the cell apoptosis process. The mRNA expression level of p53 and caspase-3 genes were increased and expression of bcl-2 gene was decreased in lung cancer cells treated with xylaranic acid and xylaranic acid AgNPs in compare to untreated cells. Results of this study revealed that p53 and caspase-3 genes are involved in the process of cell apoptosis, which is induced by xylaranic acid and xylaranic acid AgNPs. Whereas, bcl-2 proteins are involved in the inhibition of apoptosis process which is successfully decreased in treated cells^50^. Furthermore, in the apoptotic cascade p53 is a key regulator and activate the mitochondrial pathway^51–53^. However, in the process of apoptosis, apoptosome is formed due to the release of cytochrome c in to the cytoplasm from the mitochondria which convert procaspase 9 in to caspase 9. Caspase 9 is an apoptosis initiator which can later activate different apoptosis effectors like caspase 3, 6 and 7 ^46,54^. Therefore, our results indicate that xylaranic acid and xylaranic acid AgNPs may induce phosphorylation of p53 which augment the level of cytochrome c and cleaved caspase 9 which may induce lung cancer cell apoptosis via the mitochondrial pathway.

## Methods

### Sample collection, Identification and Photography

Fruiting bodies of *X*. *primorskensis* were collected with photographs from their natural habitat using sterile forceps and were packed in the sterile polyethylene bags. The fungi was identified by ITS gene sequence analysis (ITS1 and ITS2) and dried under oven at 37 °C, ground into fine powder with an electric grinder and stored in airtight bottles.

### Phylogenetics of the fungi

ITS gene sequences of fungi belongs to the genus *Xylaria* species and were downloaded from GenBank in FASTA format. Sequences were analyzed and edited by using BioEdit 7.2.5. To find out the common regions among all retrieved *Xylaria* species sequences, pairwise alignment and multiple sequence alignment (MSA) was carried out by using Clustal-W embedded in MEGA 7.0. All positions containing gaps and missing data were eliminated. Phylogenetic analyses were performed using maximum likelihood (ML) approach in MEGA 7.0.

### DNA extraction, PCR amplification and Sequencing

Total genomic DNA was extracted from the surface sterilized small inner portion of fruiting body by using method of Plaza *et al*.^55^. Quantification of DNA was done according to the method described by Sambrook *et al*.^56^. 10 μL of extracted DNA was dissolved in 30 μl of Tris buffer (pH 8) and O.D. was taken at 260 and 280 nm (PowerWave HT Microplate Spectrophotometer, BioTek). Quality was assessed by taking the (OD at 260 nm)/(OD at 280 nm). Amplification of ITS gene was carried out by using a pair of primer ITS1 and ITS4 with 1× final concentration of ReadyMix™ Taq PCR reaction mix (Sigma, India) and, template DNA (50 ng/μl). The reaction was carried out in thermal cycler (Applied Biosystems Veriti®). ITS1F 5′-TCCGTAGGTGAACCTGCGG-3′ used as a forward and ITS2R 5′- GCTGCGTTCTTCATCGATGC -3′ was used as a reverse primer^57^. PCR reaction mixture contained 1× reaction mixture (10 μL), forward primer (1 μL), reverse primer (1 μL), genomic DNA template (2 μL), and nuclease free water (6 μL). PCR program was adjusted as: 95 °C for 3 min., 35 cycles of 95 °C for 30 Sec., 55 °C for 30 Sec., and 72 °C for 1 min; and a final extension step at 72 °C for 10 min. and stored at −4 °C for ∞ time. Amplified PCR products were detected on agarose gel (1%) by electrophoresis staining with ethidium bromide and visualizing under UV light). Purification of amplified PCR product was done using GenElute™ PCR Clean-up kit. Purified PCR product was sequenced to the Eurrofins Genomics India Pvt Ltd., Banglore. The sequences were analyzed by using BioEdit 7.2.5. and subjected to sequence match analysis using Basic Local Alignment Search Tool (BLAST) on NCBI. Final edited sequences were submitted to GenBank database of NCBI.

### Extraction and identification of terpenoids

*X*. *primorskensis* powder (10 g) was soaked in absolute alcohol for 24 hours. After 24 hours, mixture was filtered, solid residues were removed and filtrate was extracted with petroleum ether up to 6 hours with frequent shaking. Later, it was treated with warm aqueous KOH (10%), two layers were separated. From that, petroleum ether layer was dried and treated as total terpenoids^33^. Extracted terpenoids were qualitatively checked through Salkowski test and HPTLC profiling. Extracted terpenoids was further subjected to silica gel column chromatography using hexane/ethyl acetate solvent system of gradually increasing polarity (0 to 100%) followed by 100% methanol. Collected fractions (7 fractions) were analysed through HPTLC profiling and similar fractions were pooled in each other. At the last, three fractions were collected. Collected three fractions were assayed against *Staphylococcus aureus* (MTCC 3160). Higher antibacterial activity was found in fraction number 3. Fraction 3 was dried at room temperature and dissolved in methanol (yield 30 mg), which was analysed as a single band through HPTLC profiling and was further purified on preparative TLC plate (silica gel 60 F_254_). Identification and structural elucidation of isolated compound was identified by comparing ^1^H and ^13^C NMR spectroscopic (FT NMR Spectrometer model advance II, Bruker) data with earlier reports^23^.

### Preparation of xylaranic acid nanoparticles

For the synthesis of AgNP, 1.5 ml of extracted xylaranic acid from the *X*. *primorskensis* was mixed with 30 ml of AgNO_3_ solution (1 mM/ml) and incubated with vigours stirring at 100 °C. Colour changes from transparent yellow to dark brown indicate the formation of Ag nanoparticles. The colour change is due to the Surface Plasmon Resonance phenomenon.

### Characterization of xylaranic acid AgNP

#### Ultraviolet-Vis Analysis

Synthesized terpenoid AgNPs were characterized by spectrophotometric analysis. Reaction mixture was centrifuged at 7000 rpm for 8 minutes and pellet was resuspended in sterile distilled water and scanned in UV-visible spectra, between 200 to 700 nm wavelengths having a resolution of 1 nm.

#### TEM analysis

Furthermore, particle size and shape of the nanoparticles were characterized by TEM. Xylaranic acid nanoparticles (1 ml) were stained with equal amount of 0.5% phosphotungistic acid solution, fixed on copper grids, dried, and then imaged with TEM in build with CCD camera (Tecnai 20, Philips, Holland).

#### FTIR analysis

The interaction between xylaranic acid – AgNPs was analyzed by FTIR in the diffuse reflectance mode using KBr pellets method (Spectrum GX, Perkin Elmer, U.S.A). Xylaranic acid was mixed with KBr powder and dried properly and subjected to measurement. Spectra were recorded in the wavelength interval of 4000-400 cm^−1^.

#### Energy Dispersive Analysis X-Ray Spectroscopy

Presence of elemental silver was confirmed through the EDS, which was carried out at the Sophisticated Instrumentation Centre for Applied Research & Testing, Anand, Gujarat, India with the model ESEM EDAX XL-30, Philips, Netherlands.

#### Screening of antibacterial property of xylaranic acid and its synthesized AgNPs

Antibacterial activity of xylaranic acid & synthesized xylaranic acid AgNPs were analysed by agar cup/well diffusion method against different pathogenic organisms like *S*. *aureus* (MTCC 3160), *S*. *typhi* (MTCC 3215) and *S*. *flexneri* (MTCC 1457) on Muller Hinton Agar (MHA) (Hi-Media, India). All the cultures were prepared by transferring a colony into a tube of nutrient broth and grown at 37 °C. Turbidity of the culture was adjusted with sterile nutrient broth to match 0.5 McFarland standards. A well was made on the plate with the help of gel puncture and 100 µl of xylaranic acid and xylaranic acid AgNPs (100 µg/ml) were inoculated into the well and plates were incubated 37 °C for 24 hours and the zones of inhibition was discussed. Gentamycin standard antibiotic was used as the positive control.

#### Determination of DPPH free radical scavenging activity

Antioxidant activity of xylaranic acid and xylaranic acid AgNP measured against DPPH in terms of radical scavenging ability^58^. Different concentration of xylaranic acid and AgNPs (25 µg/ml, 50 µg/ml, 75 µg/ml and 100 µg/ml) were added in a tube containing 2 ml of 6 × 10^−5^ M of DPPH solution in DMSO. All the tubes were incubated up to 1 hour in dark. At the end of incubation, decrease in absorbance was measured at 517 nm. DMSO was used as blank. DPPH solution without xylaranic acid and its AgNPs was used as a control. Ascorbic acid was used as a standard. All determinations were carried out in triplicate. The ability to scavenge the DPPH radical was calculated using the following equation:

$${\rm{DPPH}}\,{\rm{scavenging}}\,{\rm{activity}}( \% )=(A0-A1)/A0\times 100$$

where, A0 = absorbance of the control, A1 = absorbance of the sample.

#### Determination of hydrogen peroxide scavenging activity

Hydrogen peroxide scavenging activities of xylaranic acid and its AgNPs were measured using the method of Ruch *et al*.^59^ 1 ml of xylaranic acid and its AgNPs (25 µg/ml, 50 µg/ml, 75 µg/ml and 100 µg/ml) were mixed with a solution of H_2_O_2_ (1 ml, 2 mM) prepared in phosphate buffer (0.1 M, pH 7.4) and incubated for 10 minutes at room temperature. The absorbance was determined at 230 nm against a blank solution containing phosphate buffer without hydrogen peroxide. Ascorbic acid was used as positive control. The percentage of hydrogen peroxide scavenged was calculated using the following formula:

$${\rm{ \% }}{\rm{i}}{\rm{n}}{\rm{h}}{\rm{i}}{\rm{b}}{\rm{i}}{\rm{t}}{\rm{i}}{\rm{o}}{\rm{n}}=(({A}_{0}-{A}_{1})/{A}_{0})\times 100$$

where, A_(0)_ is the absorbance of the control, A_(1)_ is the absorbance of the extract/standard.

### Cytotoxic assay (MTT assay)

A549 cell line (lung cancer cell line) was seeded in 96-well plates at a density of more than 1 × 10^5^ cells per well and incubated in humidifier atmosphere containing 5% CO_2_ at 37 °C up to adherence. Numbers of viable cells were calculated by staining with 0.4% Trypan Blue using a haemocytometer. However, assay was performed with three repeated wells for each concentrations. Cells were then treated with different concentration of xylaranic acid and its AgNPs (20–100 µg/ml) for 24 hours. Cells were washed with PBS solution and subjected with 100 μl of MTT solution (3-(4,5-dimethylthiazolyl-2)-2,5-diphenyltetrazolium bromide) (5 mg/ml) and incubated for 4 hours. Finally, the medium was removed and 100 µl of DMSO was added to solubilise the formazan crystals. Amount of formazan crystal was determined by measuring the absorbance at 570 nm using ELISA reader. 5 – Flourouracil (5-FU) was used as a positive control. All assays were done in triplicate and 50% cytotoxic concentration (IC50) of xylaranic acid and its AgNPs were calculated.

### Determination of expression levels of apoptosis regulatory genes

Cellular RNA was isolated using the TriPure Isolation Reagent (Sigma-Aldrich, India), according to the manufacturer’s instructions. Quantification of RNA was done using 1.2% agarose gel by electrophoresis staining with ethidium bromide and visualizing under UV light^38^. 1 µg of isolated RNA was firstly reverse transcribed by RT-first strand synthesis kit (Qiagen, CA, USA). Relative expression of apoptotic genes were determined by SYBR green based qRT PCR method (Applied Biosystems 7500 Fast Real-Time PCR machine, CA, USA) and data was analyzed using ΔΔCt method and values were expressed in terms of fold change relative to control. Four pairs of primers were separately used (Table 1).

**Table 1:** List of forward and reverse primers for apoptosis regulatory genes^60^.

**Sr**. **No**	**Primer**	**Sequence**
1	p53	Forward- 5′AGAGTCTATAGGCCCACCCC3′ Reverse- 5′GCTCGACGCTAGGATCTGAC3′
2	bcl2	Forward- 5′TTCGATCAGGAAGGCTAGAGTT3′ Reverse- 5’TCGGTCTCCTAAAAGCAGGC3′
3	Caspase-3	Forward- 5′TGCGCTGCTCTGCCTTCT3′ Reverse- 5′CCATGGGTAGCAGCTCCTTC3′
4	gapdh	Forward- 5′CATGGGGAAGGTGAAGGTCGA3′ Reverse- 5′TTGGCTCCCCCCTGCAAATGAG3′

Cycling conditions for relative expression of genes were as follows: initial reverse transcription at 55 °C for 45 min, 1 cycle denaturation of 95 °C with 10 min hold, followed by 40 cycles of 95 °C with 15 s hold, annealing temperature at 60 °C (p53, bcl2, caspase-3 and gapdh) with a 60 s hold.

### Statistical analysis

All experiments were carried out in triplicate and the results are given as mean ± standard error of the mean. To compare continuous data from multiple groups, discovery determined using the two-stage linear step-up procedure of Benjamini, Krieger and Yekutieli, with Q = 1%. Each row was analyzed individually, without assuming a consistent SD. Statistical analysis was conducted with GraphPad Prism Version 7.03 software.

## Conclusion

In conclusion, this is the first study which is revealing the medicinal importance of xylaranic acid in terms of antibacterial, antioxidant and potent anticancer effect on human lung cancer cells. Moreover, novel xylaranic acid silver nanoparticle system developed in this study showed improved physico-chemical properties and increased bioactive potential of xylaranic acid. Therefore, xylaranic acid AgNPs may have potential use in the future as an effective antimicrobial solution, as natural antioxidant in the prevention of different oxidative stresses associated with degenerative diseases, as well as, in the form of treatment for lung cancer. Further results are required, detailing anti-cancer mechanism with protein expression studies to prove the authenticity of xylaranic acid AgNPs and its potential use.

## Electronic supplementary material

Supplementary Data and Figures
