# Supplementary material for: Formulation, evaluation and bioactive potential of Xylaria primorskensis terpenoid nanoparticles from its major compound xylaranic acid
Source: Sci Rep. 2018 Jan 29;8:1740. doi: 10.1038/s41598-018-20237-z (PMC5789059; doi:10.1038/s41598-018-20237-z)
Supplement: Supplementary file 1 — Supplementary Data and Figures [file 41598_2018_20237_MOESM1_ESM.pdf]

Formulation, evaluation and bioactive potential of *Xylaria primorskensis* terpenoid nanoparticles from its major compound xylaranic acid

Mohd Adnan, Mitesh Patel, Mandadi Narsimha Reddy, Eyad Alshammari

Supplementary Information

Supplementary Data S1: ^{1}H-NMR spectra and ^{13}C-NMR spectra (chemical shift values)

Xylaranic acid - light yellowish oil (MeOH). ^{1}H NMR (600 MHz, in $CDCl_3$) δ 0.92 (3H, d, J = 6.7 Hz), 1.24 (3H, s), 1.46-1.78 (4H, 1.54 (ddd, J = 13.2, 10.0, 5.8 Hz), 1.66 (ddd, J = 13.2, 1.8, 1.4 Hz), 1.74 (dd, J = 13.1, 2.3 Hz), 1.57 (dd, J = 13.1, 10.2 Hz)), 1.91 (1H, qdd, J = 6.7, 5.8, 1.4 Hz), 2.06 (1H,dddd, J = 10.2, 5.2, 3.5, 2.3 Hz), 2.38-2.58 (2H, 2.44 (dd, J = 15.2, 5.7 Hz), 2.51 (dd, J = 15.2, 8.9 Hz)), 2.95 (1H, td, J = 5.8, 5.2 Hz), 3.70-3.74 (2H, 3.72 (d, J = 5.8 Hz), 3.72 (d, J = 5.8 Hz)), 3.88 (1H, dddd, J = 10.0, 8.9, 5.7, 1.8 Hz), 4.21 (1H, dd, J = 6.8, 3.5 Hz), 5.08 (1H, d, J = 6.8 Hz). ^{13}C NMR (150 MHz, in $CDCl_3$): δ 41.9(C-1), 15.8(C-2), 152.3(C-3), 35.2(C-4), 40.9(C-5), 178.0(C-6), 36.0(C-7), 41.3(C-8), 62.3(C-9), 44.9(C-10), 120.0(C-11), 39.4(C-12), 69.0(C-13), 16.3(C-14), 68.1(C-15).

Supplementary Figure S2

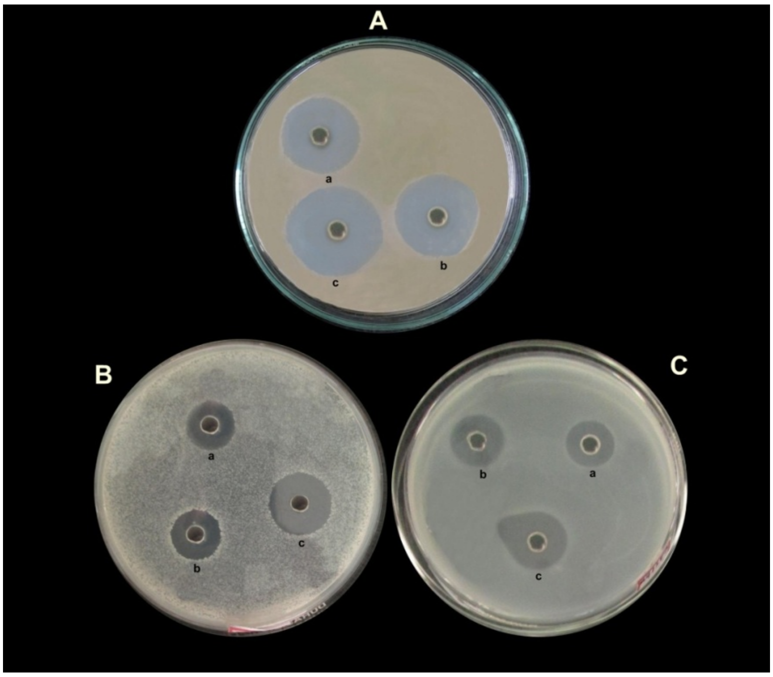

Supplementary Figure S2: Antibacterial activity of xylaranic acid and xylaranic acid AgNPs by agar cup/well diffusion method.

A) Antibacterial activity against *S. aureus* a). Positive control b). Xylaranic acid c). Xylaranic acid AgNPs

B) Antibacterial activity against *S. typhi* a). Positive control b). Xylaranic acid c). Xylaranic acid AgNPs

C) A). Antibacterial activity against *S. flexneri* a). Positive control b). Xylaranic acid c). Xylaranic acid AgNPs.

Supplementary Figure S3

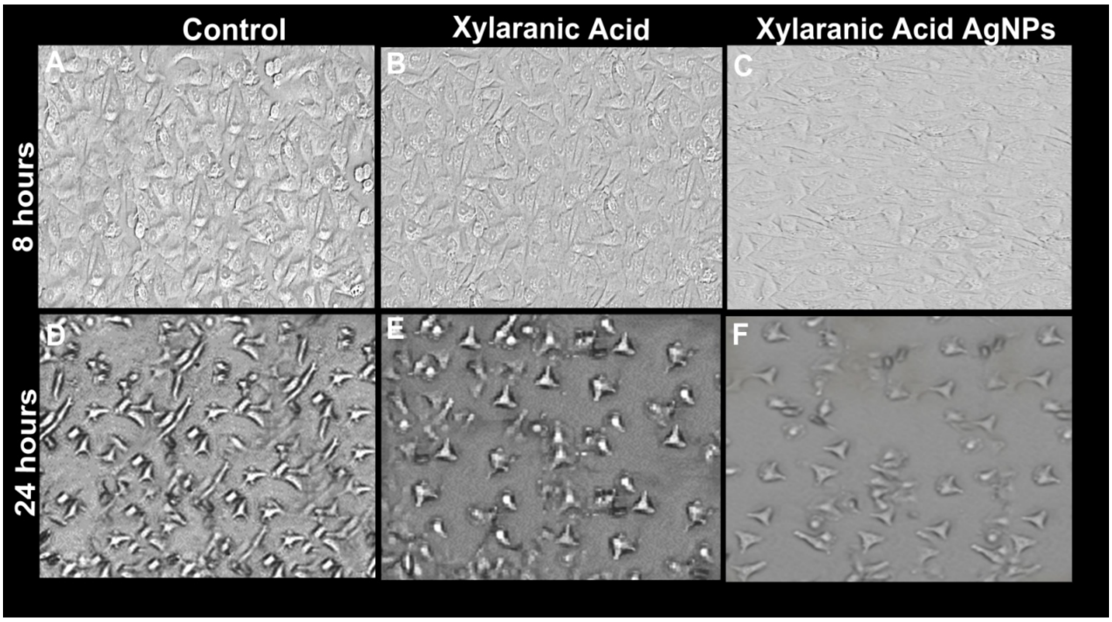

Supplementary Figure S3: The effects of xylaranic acid and its AgNPs on lung cancer cell (A549).

(A&D)- Treatment of cells with 5-flourouracil for 8 and 24 hours.

(B&E)- Treatment of cells with xylaranic acid (100 µg/ml) for 8 and 24 hours.

(C&F)- Treatment of cells with xylaranic acid AgNPs (100 µg/ml) for 8 and 24 hours.

Supplementary Figure S4

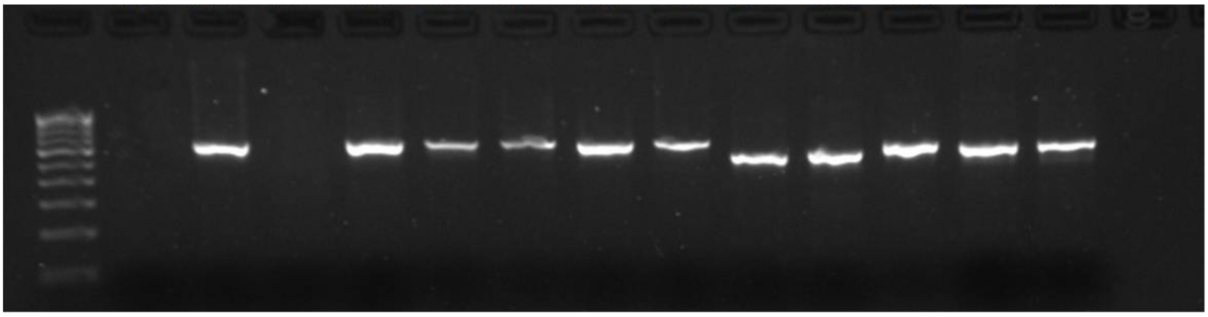

Supplementary Figure S4: Original gel image (Figure 3).
